# Inhibitor and Substrate Binding Induced Stability of HIV-1 Protease against Sequential Dissociation and Unfolding Revealed by High Pressure Spectroscopy and Kinetics

**DOI:** 10.1371/journal.pone.0119099

**Published:** 2015-03-17

**Authors:** Marek Ingr, Reinhard Lange, Věra Halabalová, Alaa Yehya, Josef Hrnčiřík, Dominique Chevalier-Lucia, Laetitia Palmade, Claire Blayo, Jan Konvalinka, Eliane Dumay

**Affiliations:** 1 Tomas Bata University in Zlín, Faculty of Technology, Department of Physics and Material Engineering, nám. T.G. Masaryka 5555, 76001 Zlín, Czech Republic; 2 Charles University in Prague, Department of Biochemistry, Hlavova 2030, 128 43 Prague 2, Czech Republic; 3 Université Montpellier 2, INRA UMR IATE, Biochimie et Technologie Alimentaires, cc023, Place Eugene Bataillon, 34095 Montpellier cedex 05, France; 4 INSERM U710, Université Montpellier 2, Place Eugene Bataillon, 34095 Montpellier cedex 05, France; 5 Université Montpellier 2, UMR IATE, Biochimie et Technologie Alimentaires, cc023, 2 Place Eugene Bataillon, 34095 Montpellier cedex 05, France; Universidad de Granada, SPAIN

## Abstract

High-pressure methods have become an interesting tool of investigation of structural stability of proteins. They are used to study protein unfolding, but dissociation of oligomeric proteins can be addressed this way, too. HIV-1 protease, although an interesting object of biophysical experiments, has not been studied at high pressure yet. In this study HIV-1 protease is investigated by high pressure (up to 600 MPa) fluorescence spectroscopy of either the inherent tryptophan residues or external 8-anilino-1-naphtalenesulfonic acid at 25°C. A fast concentration-dependent structural transition is detected that corresponds to the dimer-monomer equilibrium. This transition is followed by a slow concentration independent transition that can be assigned to the monomer unfolding. In the presence of a tight-binding inhibitor none of these transitions are observed, which confirms the stabilizing effect of inhibitor. High-pressure enzyme kinetics (up to 350 MPa) also reveals the stabilizing effect of substrate. Unfolding of the protease can thus proceed only from the monomeric state after dimer dissociation and is unfavourable at atmospheric pressure. Dimer-destabilizing effect of high pressure is caused by negative volume change of dimer dissociation of −32.5 mL/mol. It helps us to determine the atmospheric pressure dimerization constant of 0.92 μM. High-pressure methods thus enable the investigation of structural phenomena that are difficult or impossible to measure at atmospheric pressure.

## Introduction

HIV-1 protease is a small aspartic protease active as a homodimer of the molecular weight of 21.6 kDa. The dimer is stabilized by a β-sheet domain formed by the intertwined N- and C- termini of the monomers [1]. It is expressed as a part of Gag-Pol polyprotein and is autocatalytically excised from this precursor. After this release the protease plays an important role in the process of maturation of the viral particle as it cleaves the viral polyprotein precursors Gag at five sites and Gag-Pol at eleven sites. This proteolytic processing produces the structural and enzymatically active viral proteins and enables the formation of mature infectious viral particle. Inhibition of HIV-1 protease prevents viral maturation which makes the enzyme an important therapeutic target in HIV/AIDS treatment [2,3].

Stability of the HIV-1 protease dimer is an important property determining the enzyme´s activity, since only the dimeric form is enzymatically active. For quantifying its stability, many research groups have therefore conducted experiments for determining the equilibrium constant of dimerization (K_d_) of HIV-1 protease [4–7] as well as of some other retroviral proteases and HIV-1-protease mutants [7–9]. However, the obtained values varied considerably depending on the method of determination. While some groups reported extremely stable dimer characterized by subnanomolar dissociation constant K_d_ [4], other teams determined values several orders of magnitude higher [9,10].

A probable explanation of these discrepancies is the high dimer stability which leads to the necessity of measurements in very low protease concentrations at the detection limit of most analytical methods. In addition, it is not possible to observe the association of monomers directly, because an isolated monomer is impossible to prepare. Thus, when kinetics of the processes of dissociation and association of the dimer is investigated, only the first process can be directly observed, which makes the results less reliable.

High pressure methods [11–14] represent a possible way how to circumvent the experimental troubles arising from the high dimer stability. When high pressure is applied to a physicochemical system, the equilibrium is shifted towards the state with lower total volume. As there is a general experience that the volume decreases when oligomeric proteins are dissociated, it can be expected that the HIV-1 protease dimer will dissociate under high pressure. Thus, the dimer dissociation will be able to be observed at considerably higher concentration. Moreover, if the process is reversible, it should also be possible to observe the association of monomers directly after pressure release.

High-pressure techniques are increasingly used to study unfolding and oligomerization equilibrium of many proteins [15–24]. The structural changes are most often studied by variety of spectroscopic methods. However, structural distortions can result also in different set of antibodies obtained from an experimental animal after immunization by antigen treated by high pressure [25]. High pressure methods can be used also for preparative purposes, e.g. the extraction of proteins for LC-MS analysis from formaldehyde-fixed tissue samples [26]. In some cases the transition curves of dimer or oligomer dissociation were used to evaluate the volume change of this process and the atmospheric-pressure equilibrium constant [20–22]. Recently, we applied this approach to study bovine odorant-binding protein (bOBD) of the lipocalin family, which appears to share some structure-function features with HIV-1 protease. Although not enzymatically active, it is a small homodimer able of binding its ligand only in dimeric state. High-pressure methods in the range up to 600 MPa have revealed that the dissociated monomers unfold at lower pressure than a dimer without the bound ligand, while ligand binding shifts the unfolding threshold even higher [23]. Thus, dimerization stabilizes the structure of the protein which is even more stabilized by ligand binding.

Similar behaviour can be expected also in the case of HIV-1 protease. To our knowledge, however, high pressure studies with retroviral proteases have not been carried out yet. Thus, it is not known whether a reversible equilibrium of dimer and folded monomers occurs at high pressure or the dimer dissociation is directly coupled with unfolding. Recent NMR studies with mutants of HIV-1 and HIV-2 proteases with defects in dimerization domains [27,28] indicate that folded monomers can exist, but they do not allow the observation of dimerization. Another question is whether the dimer’s interaction with substrate or inhibitor stabilizes the molecule with respect to the high-pressure dissociation and unfolding. Furthermore, dimerization equilibrium constant K_d_ and the rate constants of dimer dissociation and association under high pressure have not been measured yet. Our intention is therefore to study HIV-1 protease under varying pressure in order to observe the monomer-dimer equilibrium and unfolding and to contribute to understanding its equilibrium and kinetics properties.

## Methods and Material

### HIV-1 protease expression and purification

HIV-1 protease purification procedure was modified according to [10]. It was produced by heterologous expression in *Escherichia coli* strain BL21(DE3)RIL from a gene cloned in a plasmid pET11c. Four 2L flasks with 0.5 L LB medium containing 0.1 mg/mL ampicillin were inoculated by the cells harbouring the plasmid and the cultivation was carried out until OD_600_ = 1.0. The protein expression was then induced by the addition of 0.25 mM Isopropyl β-D-1-thiogalactopyranoside (IPTG) and cultivated for 3 h. Cells were harvested by centrifugation, washed by PBS buffer, resuspended in buffer A (50 mM Tris, 50 mM NaCl, 1 mM EDTA, pH 8.0) and subjected to three freeze-thaw cycles. Cells were further disintegrated by sonication in five 1-min cycles on ice. Cell debris was centrifuged for 30 min, 15000 g, 4°C, the supernatant was removed and the peleted inclusion bodies were resuspended in buffer SA (A + 1 M NaCl) and sonicated in three 1-min cycles on ice. After centrifugation for 20 min, 15000 g, 4°C the previous washing procedure was repeated in TA buffer (A + 1% Triton X-100). One half of the inclusion bodies were resuspended in 2 mL of water and dissolved in 4 mL of 99% acetic acid, then it was diluted 30 times by 0.05% 2-mercaptoethanol in water and dialyzed against 2 L of the same solution. The dialysis continued against three 2L pools of buffer 20 mM Tris, 20 mM MES, 10% glycerol, 0.05% 2-mercaptoethanol, 1 mM EDTA, pH 6.7. Precipitate was removed by centrifugation for 10 min, 15000 g, 4°C and the supernatant was purified by cation-exchange chromatography on Capto S column (GE healthcare) with the binding buffer CA equal to the dialysis buffer and the elution buffer CB (CA + 1 M NaCl). Fractions eluted by the 30-min gradient of 0–100% CB containing the protease were pooled and further purified by gel permeation chromatography on Sephacryl S-100 16/60 (GE Healthcare) in buffer 20 mM CH_3_COONa, 200 mM NaCl, 10% glycerol, 0.05% 2-mercaptoethanol, 1 mM EDTA, pH 5.0 (hereafter called “assay buffer”). Fractions containing the protease were pooled and concentrated on Amicon centrifugation cell with cut-off 10 kDa to the final value of about 0.5 mg/mL.

### Theoretical description of the structural transitions

#### Monomer-dimer equilibrium at high pressure

At given temperature and pressure the equilibrium between dimer (D) and monomer (M)

D⇄2M (1)

is described by an equilibrium constant K_d_

$$K_{d}\left(p\right)=\frac{{\left[M\right]}^{2}}{\left[D\right]},$$ (2)

Where [*M*] and [*D*] are the relative molar concentrations of monomer and dimer, respectively. If the total concentration of the protein expressed as monomer is *M*_0_, the equilibrium monomer concentration is

$$\left[M\right]=\frac{-K_{d}+\sqrt{K_{d}{}^{2}+8K_{d}\left[M_{0}\right]}}{4}.$$ (3)

Under the varying pressure the equilibrium constant takes the form

$$K_{d}\left(p\right)=e^{\frac{-\Delta G_{d}^{0}\left(p\right)}{RT}}=e^{\frac{-\left(\Delta G_{d,atm}^{0}+\Delta V_{d}\Delta p\right)}{RT}}=K_{d,atm}e^{\frac{-\Delta V_{d}\Delta p}{RT}},$$ (4)

where $\Delta G_{d}^{0}\left(p\right)$ is the standard molar reaction change of Gibbs energy at certain pressure *p* and $\Delta G_{d,atm}^{0}$ is the same quantity at atmospheric pressure, *K*_*d*,*atm*_ is the atmospheric pressure equilibrium constant and Δ*V*_*d*_ is the volume change of dimer dissociation which is considered to be constant within the whole pressure range. The symbol Δ*p* stands for the difference of a certain pressure and atmospheric pressure.

Combining Eqs. (3) and (4) the expression for monomer concentration at given [*M*_0_] and Δ*p* is obtained

$$\left[M\right]=\frac{K_{d,atm}e^{\frac{-\Delta V_{d}\Delta p}{RT}}}{4}\left(-1+\sqrt{1+\frac{8\left[M_{0}\right]}{K_{d,atm}e^{\frac{-\Delta V_{d}\Delta p}{RT}}}}\right).$$ (5)

Introducing the degree of dimer dissociation, or rather a monomer fraction, *α*_*d*_ = [*M*]/[*M*_0_] Eq. (3) can be expressed as

$$\alpha_{d}\left(\Delta p\right)=\frac{K_{d,atm}e^{\frac{-\Delta V_{d}\Delta p}{RT}}}{4\left[M_{0}\right]}\left(-1+\sqrt{1+\frac{8\left[M_{0}\right]}{K_{d,atm}e^{\frac{-\Delta V_{d}\Delta p}{RT}}}}\right),$$ (6)

which defines a sigmoid-like curve with the limits of 0 and 1 for [*M*_0_] tending to infinity and zero, respectively. After some algebraic rearrangements the position of inflex point of this curve can be derived:

$$\Delta p_{\text{inf}}=\frac{-RT}{\Delta V_{d}}\text{ln}\frac{4\left[M_{0}\right]}{K_{d,atm}\left(1+\sqrt{2}\right)}=\frac{-RT}{\Delta V_{d}}\ln\frac{4\left[M_{0}\right]}{\left(1+\sqrt{2}\right)}-\frac{\Delta G_{d,atm}^{0}}{\Delta V_{d}}$$ (7)

Thus, Δ*p*_inf_ can be used to determine the atmospheric-pressure equilibrium constant of dimerization *K*_*d*,*atm*_, (or equivalently $\Delta G_{d,atm}^{0}$) and the molar change of volume Δ*V*_*d*_, provided that the dependence of Δ*p*_inf_ on [*M*_0_] described by Eq. (7) is known at least for two different total enzyme concentrations [*M*_0_]. Measuring this curve for a series of concentrations, *K*_*d*,*atm*_ and Δ*V*_*d*_ can be determined either from non-linear regression of this equation or with the aid of the linearized formula

$$\Delta p_{\text{inf}}\left(\left[M_{0}\right]\right)=\frac{-RT}{\Delta V_{d}}\text{ln}\left[M_{0}\right]+\frac{RT}{\Delta V_{d}}\text{ln}\frac{\left(1+\sqrt{2}\right)K_{d,atm}}{4},$$ (8)

Where $\frac{-RT}{\Delta V_{d}}$ is the slope and $\frac{RT}{\Delta V_{d}}\text{ln}\frac{\left(1+\sqrt{2}\right)K_{d,atm}}{4}$ is the Δ*p*_inf_-axis intercept of the resulting line. The slope of the curve in the inflex point is then given by

$$\frac{\partial \alpha_{d}}{\partial p}|{}_{\Delta p_{\text{inf},d}}=\frac{\Delta V_{d}}{RT}\left(2\sqrt{2}-3\right).$$ (9)

#### Monomer folding-unfolding equilibrium and kinetics

For this process described by an equation

$$M{\;}_{\underset{k_{f}}{\leftarrow }}^{\overset{k_{u}}{\to }}\;U,$$ (10)

Where *U* denotes the unfolded monomer and *k*_*u*_ and *k*_*f*_ are the rate constants of unfolding and folding processes, respectively, the inflex point of the dependence of degree of unfolding *α*_*u*_ = (*M*_0_ − *M*)/*M*_0_ is given by the expression

$$\Delta p_{\text{inf},u}=-\frac{\Delta G_{u,atm}}{\Delta V_{u}}=\frac{RT}{\Delta V_{u}}\text{ln}\;K_{u,at}$$ (11)

and the slope at the inflex point is

$${\frac{d\alpha_{u}}{d\Delta p}|}_{\Delta p_{\text{inf},u}}=-\frac{\Delta V_{u}}{4RT},$$ (12)

Where Δ*V*_*u*_ is the volume change of this process considered as pressure independent, Δ*G*_*u*,*atm*_ is the change of the Gibbs energy of unfolding at atmospheric pressure and *K*_*u*,*atm*_ is the atmospheric pressure equilibrium constant. The rate constants *k*_*u*_ and *k*_*f*_ were calculated based on the determined equilibrium parameters and the observed relaxation rate constant of the unfolding

$$k_{obs}=k_{f}+k_{u}$$ (13)

$$k_{u}\left(p\right)=k_{obs}\frac{K_{u}\left(p\right)}{K_{u}\left(p\right)+1}$$ (14)

$$k_{f}\left(p\right)=k_{obs}\frac{1}{K_{u}\left(p\right)+1},$$ (15)

where

$$K_{u}\left(p\right)=K_{u,atm}e^{-\frac{\Delta V_{u}\Delta p}{RT}}.$$ (16)

### High pressure fluorescence experiments

Fluorescence measurements were carried out at 25°C using an SLM Series 2 spectrofluorimeter (Aminco Bowman, Foster City, CA), modified to accommodate a thermostated high pressure optical cell. HIV-1 protease was diluted to various concentrations (1 to 25 μM dimer) in the assay buffer. For some experiments, inhibitor darunavir (120 μM) or 8-anilino-1-naphthalenesulfonate (ANS) (700 μM) was added to HIV-1 protease solutions. The sample was placed in a 5 mm diameter quartz cuvette, closed at the top with a flexible polyethylene film that was attached by a rubber O-ring.

#### Pressure dependence of tryptophan fluorescence spectra

Emission spectra of both inhibited and non-inhibited protease were measured at the excitation wavelength of 280 nm (slit of 4 nm) in the emission-wavelength range of 300–400 nm (slit of 8 nm). A sample of HIV-1 PR was prepared diluting the stock solution by the assay buffer to the desired concentration from the range of 1 to 25 μM dimer, pipetted into the quartz cuvette and placed in the instrument. Initial pressure of 10 MPa was set, the sample was incubated for 3 min and then the fluorescence spectrum was recorded in triplicate and averaged. Afterwards the pressure was set to the next value and the procedure was repeated. A typical pressure series consisted of pressures of 10, 25 and 50 MPa and further pressures increasing by 25 MPa up to 500 MPa, in some cases 600 MPa, excitation wavelength was 280 nm and the emission wavelength range was 300–400 nm. Centre of spectral mass (CSM) and spectral integral (SI) defined as

$$SI=\int_{\lambda_{i}}^{\lambda_{f}}F\left(\lambda \right)d\lambda$$ (17)

$$CSM=\frac{\int_{\nu_{i}}^{\nu_{f}}\;\nu \;F\left(\nu \right)d\nu }{\int_{\nu_{i}}^{\nu_{f}}\;F\left(\nu \right)d\nu }.$$ (18)

were determined for every pressure point giving the dependencies of these quantities as functions of pressure. *F*(*λ*) is the fluorescence intensity at the wavelength *λ* while *λ*_*i*_ and *λ*_*f*_ denote the lower and upper boundary of the spectral range and $\tilde{\nu }$, ${\tilde{\nu }}_{i}$ and ${\tilde{\nu }}_{f}$ are the respective wavenumbers corresponding to the wavelengths by the relations $\tilde{\nu }=c/\lambda$ and analogous. In addition, a set of differential spectra was calculated for every concentration. A spectrum for a given pressure was normalized to the spectral integral of the spectrum at 10 MPa and the 10-MPa spectrum was subtracted from it.

#### Determination of dimerization equilibrium constant and volume change

Experimentally determined curves of CSM vs. pressure were fitted by model functions of a sigmoidal shape in the form of

$$F\left(\Delta p\right)=I_{0}-q\Delta p+\frac{I_{f}-I_{0}+\left(q-m\right)\Delta p}{1+e^{-\frac{\Delta G+\Delta p\Delta V}{RT}}}$$ (19)

Where *I*_0_, *I*_*f*_, Δ*G* and Δ*V* are variable parameters of the fit and *q* and *m* were set firmly as a limit of the slope at p → −∞ or +∞, respectively. Eq. (19) was used as a suitable fitting function allowing the determination of inflex points, although in case of second-order transition the parameters Δ*G* and Δ*V* do not have the meaning of Gibbs energy and volume changes. Direct non-linear regression by a function based of Eq. (6) was avoided as it is unstable due to its complexity and many unknown parameters. An inflex point of the model functions was determined as

$$\Delta p_{\text{inf}}=-\frac{\Delta G}{\Delta V}$$ (20)

and plotted vs. logarithm of concentration providing a linear dependence given by Eq. (8). The values of Δ*V*_*d*_ and *K*_*d*,*atm*_ were obtained by linear regression of the experimental points.

#### Pressure-jump kinetics

A sample of a selected concentration was prepared and the pressure was set to 10 MPa. After 3 min incubation the pressure was set to 50 MPa by a sudden increase of pressure and the fluorescence was recorded for hundreds to thousands of seconds. The dead time of the pressure jump was less than 5 ms. The excitation and emission wavelengths were 280 nm and 350 nm, respectively. This procedure was repeated for a series of pressures increasing by 50 MPa up to 450 MPa. The whole sequence was performed for the dimer concentrations of 1, 2, 5, and 20 μM. The obtained curves were fitted by the single-exponential decay function and the equilibrium values of fluorescence as well as the apparent rate constants were determined. For every concentration the pressure dependence of the equilibrium fluorescence was fitted by the sigmoidal function given by Eq. (19) with the parameters Δ*G* and Δ*V* renamed to $\Delta G_{u,atm}^{0}$ and Δ*V*_*u*_ denoting the atmospheric pressure standard change of Gibbs energy and the volume change of the unfolding process, respectively. Furthermore, the values of rate constants of unfolding and folding of the protease monomers were calculated for every pressure point using Eqs. (14, 15).

#### Reversibility assay

A sample of 5 μM (considered as dimer) HIV-1 PR in the assay buffer was prepared, incubated for 3 min at 10 MPa in the cuvette space of the fluorometer and the tryptophan-emission spectrum was recorded as previously. Then the pressure was set to the selected target pressure, the sample was incubated for 50 min and another spectrum was recorded. The pressure was then released back to 10 MPa and the final spectrum was measured after 3 min incubation. CSM of each spectrum was evaluated. The experiment was repeated for several target pressures from the interval of 100–350 MPa. In a parallel experiment the incubation time was shortened to 3 min.

#### ANS-indicated transition

700 μM 8-anilino-1-naphtalenesulfonic acid (ANS) was added to the sample of HIV-1 PR of a given dimer concentration (2, 5, 12, and 20 μM) in the assay buffer. The sample was pre-incubated in the fluorometer pressure cell at 10 MPa for 5 min. Emission spectrum was recorded in triplicate, excitation wavelength was 350 nm and emission-wavelength range 400–600 nm. The procedure was repeated for a series of pressures up to 500 MPa with typical increase of 40–50 MPa. At the pressure points where the spectrum was obviously changing the measurement was repeated several times with 4-min incubation periods between the two subsequent measurements.

#### Kinetic experiments with ANS

In the first experiment, the sample was prepared identically as in the previous paragraph and the pressure-jump experiment was carried out in the same way as in case of the tryptophan spectra with the excitation and emission wavelengths of 350 nm (slit of 2 nm) and 472 nm (slit of 8 nm), respectively. In the second experiment whole spectra were measured in a series of time points after each pressure jump and the CSM was evaluated for every spectrum.

### High-pressure enzyme-kinetics experiments

Absorbance kinetic experiments were carried out using a Cary 3 spectrophotometer (Foster City, CA) modified to accommodate a high pressure cell. Chromogenic peptide substrate of the sequence KARVNle*NphEANle-NH_2_ (Nph stands for p-nitrophenylalanine, Nle for norleucine, asterisk indicates the cleavage site) with known kinetics parameters (Km = 15.1 μM; kcat = 30.0 s^−1^ [29,30]) was used. The volume of 2900 μL of the reaction buffer were mixed with 60 μL of substrate at 3.95 μM. The mixture was equilibrated in a water bath at 25°C for 17 min before adding the enzyme. 40 μL of enzyme (diluted by reaction buffer to the final concentration of 1 μM considered as dimer) were promptly introduced at the mixture and the sample was transferred immediately to the high-pressure cell of the instrument. As this manipulation requires 2–4 min, the concentrations were chosen in order that the linear decrease of substrate concentration takes considerably longer time. The excitation wavelength was set to 305 nm (2 nm slit width) and the absorbance was recorded for 20 min under the varying pressure in the region of 10–350 MPa at 25°C. The measured reaction rate was then normalised to the initial value at 10 MPa and plotted vs. pressure.

## Results

### High pressure treatment of HIV-1 protease complex with inhibitor darunavir

**Fig 1:**
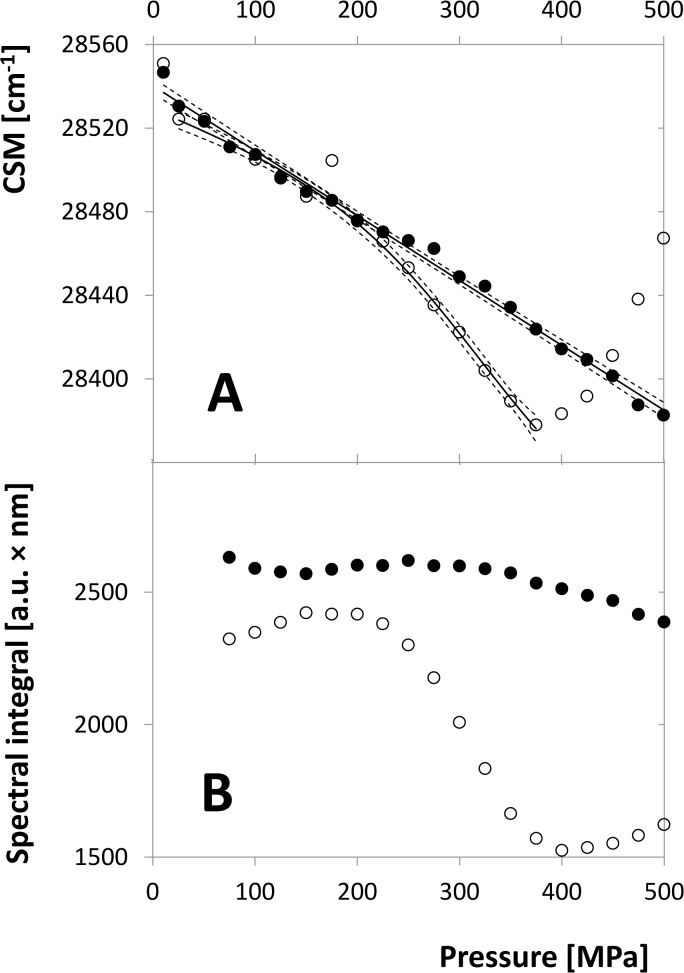
Spectral properties of inhibited (solid circles) and non-inhibited (open circles) HIV-1 PR as functions of pressure. A. CSM of the tryptophan fluorescence spectrum, λ_ex_ = 280 nm, λ_em_ = 300–400 nm. Curves are mutually shifted by an additive constant in order to allow the comparison of their shape details. Solid lines represent the regression curves of the data series (linear for inhibited and sigmoidal according to Eq.19 for non-inhibited curve) and dashed lines the corresponding confidence bands. As in Fig. 3, points of the non-inhibited series above 375 MPa, which are influenced by aggregation, are excluded from the regression procedure. B. Spectral integral of the same spectra.

HIV-1 protease solution of 12 μM dimer was mixed with 120 μM darunavir and the pressure was increased stepwise from the lowest value of 10 MPa to the maximum of 500 MPa recording the fluorescence spectrum in every pressure point. Fig. 1 shows the comparison of pressure dependences of CSM and spectral integral for inhibited and non-inhibited enzyme. It can be seen that the spectral integral change of the inhibited enzyme is relatively small in comparison with the strong sigmoid-like decrease of the free-enzyme curve. Accordingly, the CSM curve of the inhibited-enzyme decreases almost linearly, while the free-enzyme curve follows this trend only at the beginning of the pressure range up to approximately 200 MPa and then diverges from the inhibited curve to lower values. This trend is then reversed at 375 MPa, from where the curve goes steeply up. The continuous CSM decrease common for both the curves is likely an expression of a direct influence of the high pressure to the fluorophores as was demonstrated by [31] on free tryptophan and its derivatives. Its slope is equal for both the curves in the pressure range where the free enzyme can be expected to stay in the unperturbed dimeric form. Hence, the “inhibited” curve shows neither the characteristic sigmoidal shape indicating a conformation transition nor any other substantial variations of the trend, while the “free” curve does. Thus, both CSM and spectral intensity indicate that the structure of the enzyme is stabilized by the inhibitor within the chosen pressure range. This conclusion is supported also by the differential spectra shown in S2 Fig. (see the next section).

Similar experiments were carried out also for the dimer concentration of 2 μM with two different concentrations of the inhibitor, 120 μM and 6.7 μM. In accord with the previous experiment, obviously changing trend of the free-enzyme CSM curve can be seen while the inhibited curve is linear (data not shown).

### Dimerization equilibrium studied by high-pressure fluorescence spectroscopy

**Fig 2:**
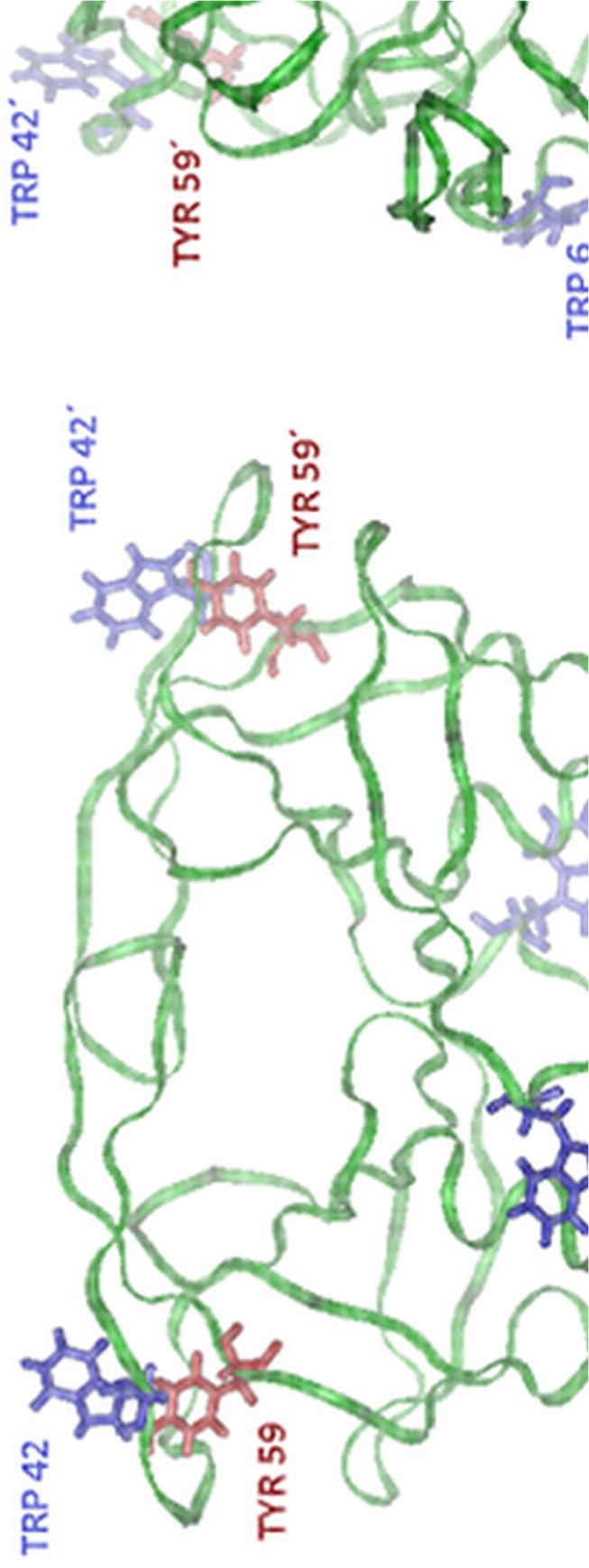
Model of the HIV-1 PR backbone in front (left) and side (right) view (structure taken from RSCB protein data bank, PDB ID: 1OHR). The residues of fluorescent amino acids, Trp6, Trp42 and Tyr59, are indicated. Trp6 is located close to the dimerization interface, therefore its spectroscopic properties are probably influenced by dimer dissociation. On the contrary, Trp42 and Tyr59 are located at the most distant site from the dimerization interface, therefore their fluorescence is probably unaffected by dimer dissociation, provided that the monomer keeps its conformation. Trp42 and Tyr59 can eventually partially influence this effect due to the change of their mutual configuration because the conformation of this part of the molecule undergoes partial changes with growing pressure, as was shown by the molecular-dynamics simulation [32]. All the fluorophores can contribute to the spectral changes induced by unfolding of monomers or protein aggregation when they undergo more extensive changes of their environment.

**Fig 3:**
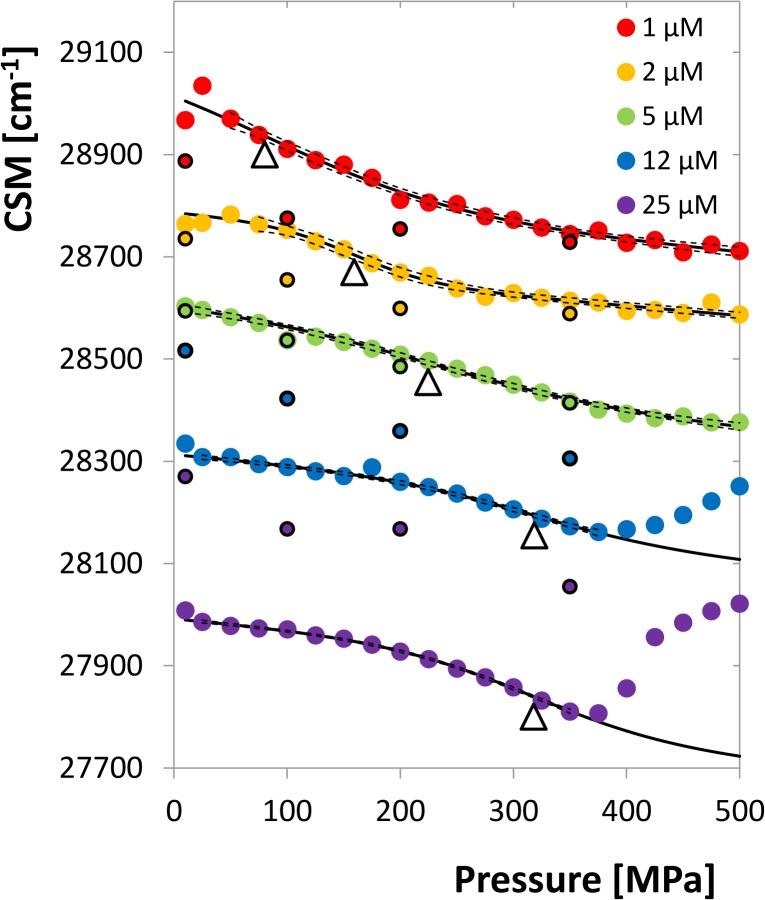
Centre of spectral mass of the emission spectra as a function of pressure for different HIV-1 protease concentrations. The curves are shifted up or down by artificial additive constants for the sake of lucidity. Solid lines express the fitting curves, empty triangles indicate the inflex points. Dashed lines represent the 95% confidence bands of the regression curves. The high pressure regions of the curves for 12 and 25 μM dimer concentration are excluded from fitting as they are influenced by protein aggregation. The smaller circles with black bordering line indicate the CSM values for the subsequent release of pressure from the highest to the lowest value.

Spectral response of non-inhibited HIV-1 protease to high pressure was determined for a dimer concentration range of 1 to 25 μM. The structure of this enzyme with the location of the fluorescently active amino acids is shown in Fig. 2. For every concentration point tryptophan emission spectra were measured in the range of 10 to 500 MPa and the pressure dependences of CSM were evaluated. A set of emission spectra for selected pressures is shown in S1 Fig. The experiment was carried out in two series, the resulting curves of one of them are shown in Fig. 3. As can be seen, the curves show the typical sigmoidal profile characteristic for structural transitions.

Differential-spectra sets calculated for different protease concentrations are shown in S2 Fig. In the presence of the inhibitor all the curves show equal shape and differ only by their amplitude, likely as a consequence of the direct pressure influence on the fluorophores. Without the inhibitor the positions of the extremes of the spectra move with the varying pressure indicating more complex structural changes which is in agreement with Fig. 1A.

**Fig 4:**
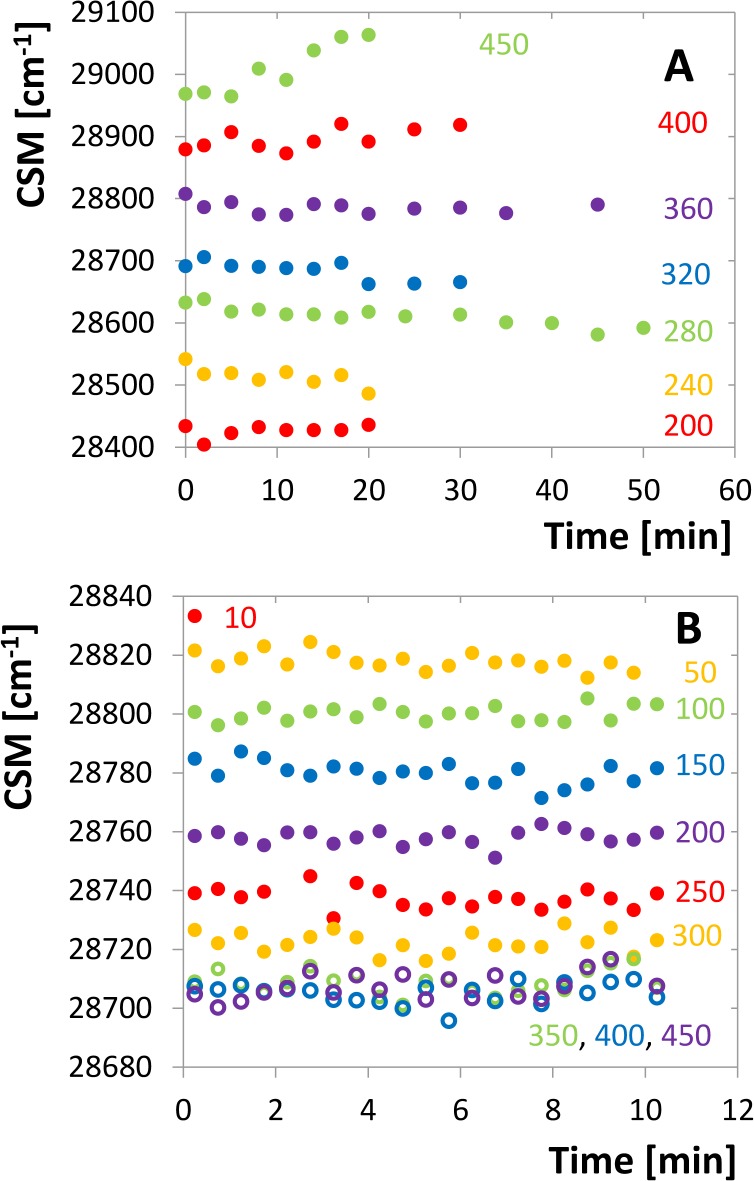
Time development of the tryptophan-fluorescence CSM. A. Time series for different pressures, each started with a new enzyme sample, concentration of 10 μM dimer. The individual curves are shifted up or down by artificially chosen constant for the sake of better orientation in the graph. B. Time series measured with the same sample of 2 μM concentration, pressure setting is facilitated by pressure jumps. The CSM scale is genuine for all the series.

To confirm that the CSM at a given pressure does not change with time, two different kinetics experiments were carried out. In the first one a sample of the enzyme was put into the pressure cell and the pressure was set manually to the desired value from the range of 200 to 450 MPa. Then the tryptophan emission spectra were taken in a series of time points and the CSMs were determined. Fig. 4A shows that no significant CSM change can be observed at any of these curves. Up to 360 MPa slightly decreasing tendency can eventually be seen the magnitude of which is considerably lower than the CSM changes induced by the pressure increase. More significant increase can be observed at very high pressures above 400 MPa which can be associated with aggregation of the protein (see the description of this phenomenon below).

In addition, a pressure-jump kinetic experiment was carried out providing the time dependence of the emission spectrum for every pressure point. In accord with the previous experiment the CSM position is stable and does not vary within the time course (Fig. 4B). Both these experiments demonstrate that the pressure dependence of CSM refers to a fast structural transition reaching its equilibrium in the time scale shorter than the experimental response, i.e. the order of magnitude of the half-times of the observed processes is surely lower than a second.

**Fig 5:**
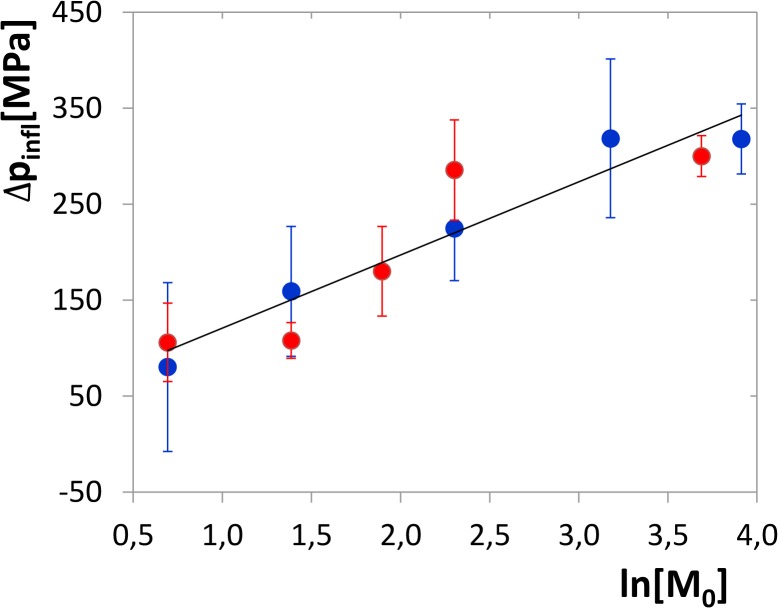
Concentration dependence of the inflex point of the CSM curves. The data consist of two independent experimental series (blue and red circles). Linear-regression line of the slope of 76.3 MPa and intercept of 45 MPa is shown which is used to determine the constants *K*_*d*,*atm*_ = 0.92 *μM* and Δ*V*_*r*_ = 32.5 *ml mol* ^−1^. Regression lines of the individual series are not shown as they are almost identical; their slopes and intercepts are 77.3 MPa and 43 MPa, respectively (blue), and 74.7 MPa and 47 MPa, respectively (red), which corresponds with *K*_*d*,*atm*_ = 0.95 *μM*, Δ*V*_*r*_ = 32.1 *ml mol* ^−1^ (red) and *K*_*d*,*atm*_ = 0.88 *μM*, Δ*V*_*r*_ = 33.2 *ml mol* ^−1^ (blue). The error bars of the individual measurements are calculated from the errors of the regression parameters of Eq. 19 using Eq. 20 and the error-transition law. [*M*_0_] is considered to be a dimensionless quantity related to the units of μM ([*M*_0_] → [*M*_0_]/*μM*).

The inflex points of the CSM curves apparently shift to higher pressures with increasing concentration, which is characteristic for transitions where the reaction volume decreases along with dissociation of multisubunit complexes, e.g. dimer dissociation. In addition, the limits of the curves at plus and minus infinity have non-zero, but practically equal slope corresponding to that of the inhibited curves. The curves of the two highest concentrations turn steeply up above 350 MPa, likely due to the protein aggregation (see below). This part of the curves was therefore excluded from the evaluation of the dimerization parameters. Inflex points of all the curves were determined and plotted vs. logarithm of monomer concentration, as shown in Fig. 5. Dimer-dissociation volume change Δ*V*_*d*_ = (−32.5 ± 4.1)*ml mol* ^−1^ and the atmospheric-pressure equilibrium constant *K*_*d*,*atm*_ = (0.92 ± 0.17)*μM* were evaluated by means of linear regression in accord with Eq. (8). The ratio of the volume change to molar weight of HIV-1 PR (−1.50 ml/kg) is in a reasonable agreement with previously studied proteins yeast enolase (−1.28 ml/kg) [20] or yeast hexokinase (from −1.07 to −1.49 ml/kg) [21].

**Fig 6:**
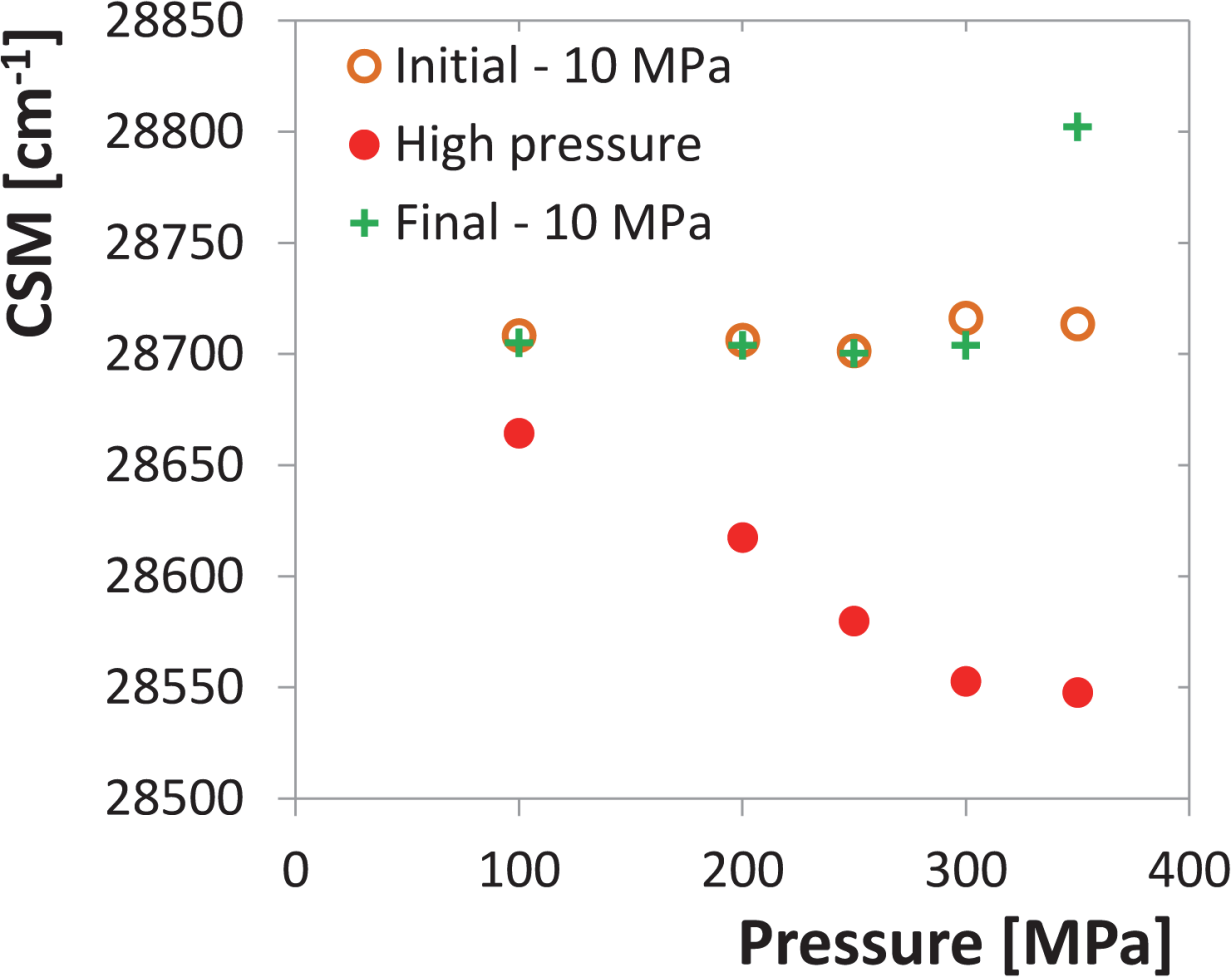
Reversibility assay of HIV-1 PR of 5 μM concentration (expressed as dimer). For every enzyme sample three CSM values were determined: at 10 MPa before pressurizing, at the selected target pressure from the interval of 100 to 350 MPa after 50 min incubation and again at 10 MPa after releasing the pressure. The experiment indicates good reversibility of CSM up to 300 MPa, above this value the reversibility is perturbed by protein aggregation.

In order to confirm the reversibility of the dimer dissociation, reversibility assay was carried out. Here, spectra are recorded at 10 MPa, then after 50 min incubation at the target pressure and again at 10 MPa after the pressure release. As shown in Fig. 6, CSM values follow approximately the sigmoid-like curves shown in Fig. 3 for the target-pressure spectra and return to the original value after the pressure release. Similar experiment, but with missing the 50 min incubation period, is shown in S3 Fig. At 300 MPa the final value after the incubation period is a little lower than the initial one, which is not observed without incubation. This might be a consequence of the folding kinetics, but the difference is quite small and should be taken with caution. However, at 350 MPa a partial contribution of the protein aggregation shifts up both the high pressure value and the value after releasing the pressure in the experiment with the 50 min incubation period, which is apparently different from the incubation-free. Thus, in accord with the transition curves shown in Fig. 3, the dimerization reflected by the CSM transition seems to be a reversible process in the pressure interval of 10–350 MPa.

### Unfolding of monomers influences the fluorescence intensity

**Fig 7:**
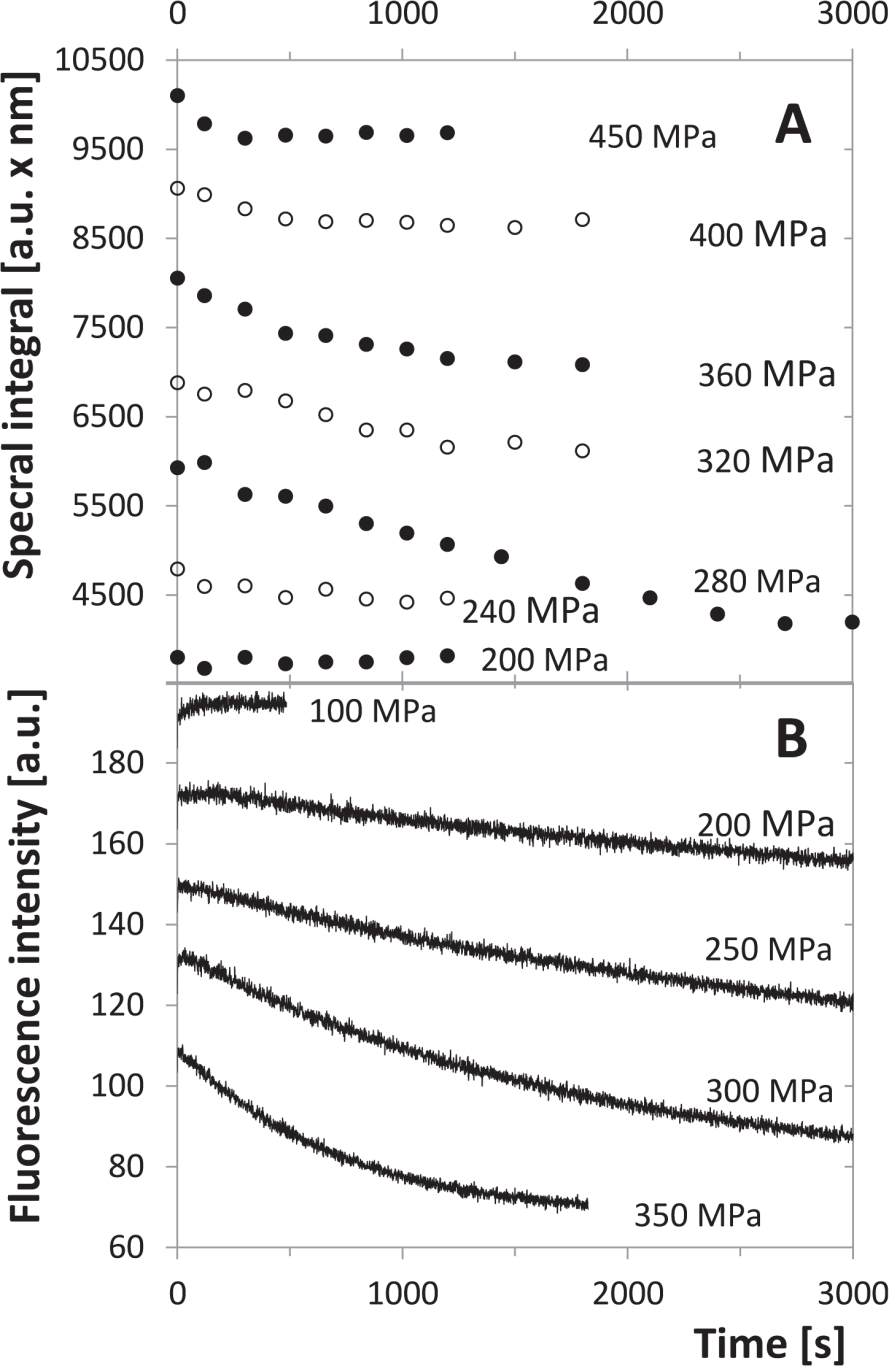
Time development of the emission intensity of the tryptophan fluorescence at λ_ex_ = 280 nm. In both panels the curves are shifted up or down by artificially chosen constant for better orientation in the graph. A. Spectral integral of spectra taken in time series for different pressures, each series with new protease sample, λ_em_ = 300–400 nm, dimer concentration 10 μM. B. Emission intensity at a single wavelength of λ_em_ = 350 nm for the dimer concentration of 5 μM.

**Fig 8:**
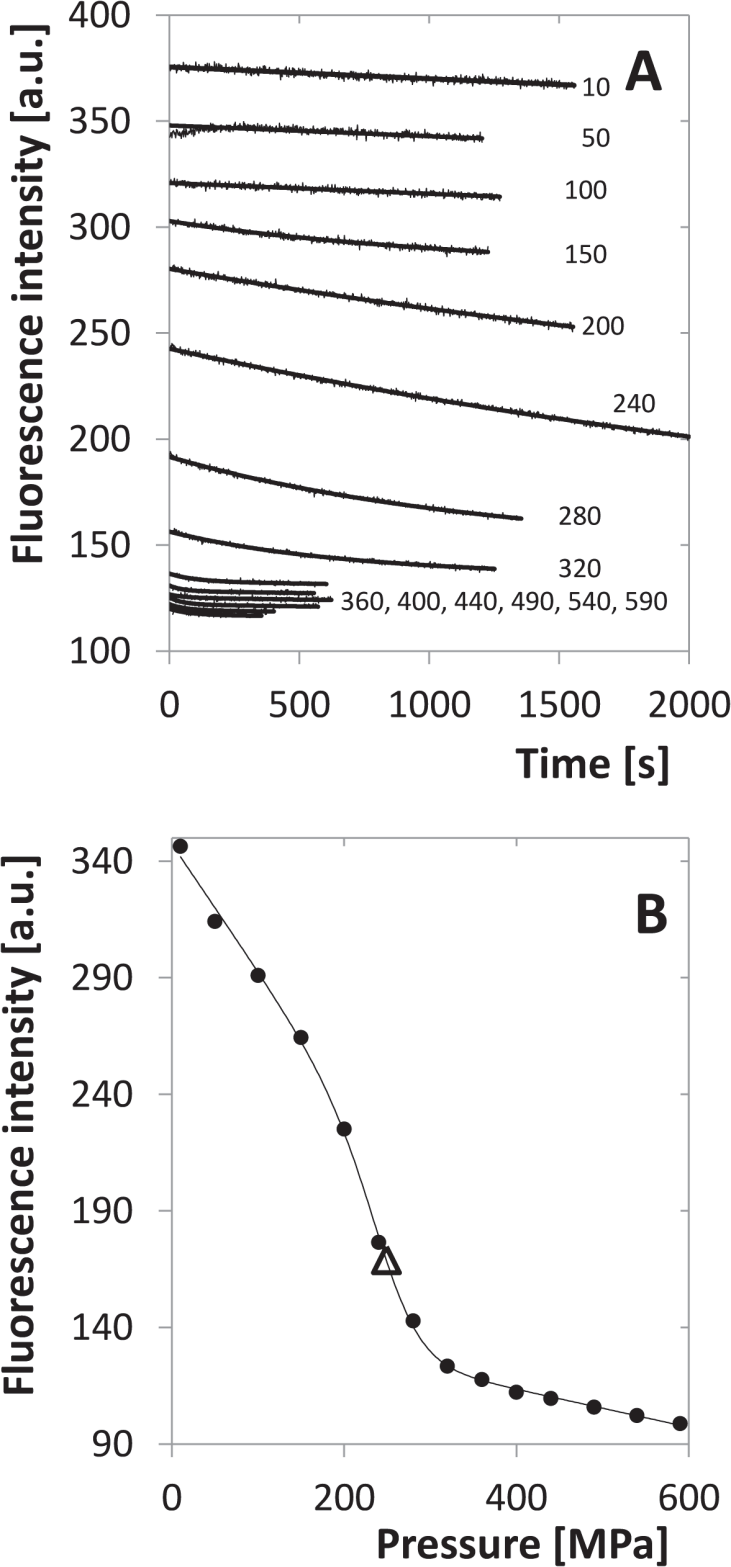
Fluorescence-intensity indicated transition for 5 μM dimer. A. Time dependence of the intensity for different pressures. Each series is plotted together with the fitting single-exponential decay curve. The same sample was used for the whole set of measurements, pressure setting was facilitated by pressure jumps. The numbers indicate pressure in MPa. B. Equilibrium values of fluorescence for the same experimental series determined from the limit of the fitting curves for time tending to infinity. Inflex point is indicated by the open triangle.

In addition to CSM, spectral intensity of the measured spectra was studied, too. The curves representing the pressure dependence of the spectral integral look qualitatively similar to the CSM curves (see the non-inhibited curves in Fig. 1), but they vary for quite a long time period. Therefore, a series of kinetic experiments was carried out in order to determine the time dependence of the spectral intensity at different pressures and concentrations. The measured curves (Fig. 7) show an obvious decrease within the time course, the magnitude of which depends on the pressure. A series of pressure-jump experiments with varying enzyme concentrations was performed. A typical set of the measured intensity curves including the fitting single-exponential functions is presented in Fig. 8A. The pressure dependent fluorescence intensity changes were not fully reversible. However, at the end of each kinetics, no further time dependent changes occurred, allowing the determination of apparent thermodynamic parameters. The apparent equilibrium values of fluorescence were evaluated for each curve and plotted as functions of pressure (an example is given in Fig. 8B). These functions have a sigmoid-like profile typical for the structural transitions. The values of the apparent volume change Δ*V*_*u*_ and atmospheric pressure Gibbs-energy change $\Delta G_{u,atm}^{0}$ of this transition were determined by means of non-linear regression. They are listed in Table 1 and show relatively high stability with respect to the varying concentration, which indicates a transition of first-order kinetics in both directions. Thus, this transition represents presumably an unfolding of the protease monomers. $\Delta G_{u,atm}^{0}$ of the series with the highest concentration is somewhat higher in comparison with the other concentrations which might be a consequence of preventing the unfolding by dimer formation.

**Table 1:** Equilibrium properties of monomer unfolding indicated by intensity of the tryptophan fluorescence spectrum.

Enzyme concentration(expressed as dimer) [μM]	*Δp*_*infl*_ [MPa]	*ΔG*_*u*_^*0*^ [kJ.mol^−1^]	*ΔV*_*u*_ [mL.mol^−1^]	*ΔV*_*u*_^‡^ [mL.mol^−1^]	*ΔV*_*f*_^‡^ [mL.mol^−1^]	*k*_*u*, *atm*_ [*s* ^*−1*^]	*k*_*f*, *atm*_ [*s* ^*−1*^]
**1**	252 ± 15	21.3 ± 1.2	−85 ± 4				
**2**	267 ± 51	29.6 ± 3.8	−111 ± 14	−47.5	63.2	0.014	2153
**5**	249 ± 54	23.0 ± 4.0	−92 ± 15	−74.9	23.5	8.4×10^−4^	8.9
**20**	288 ± 45	36.4 ± 3.4	−126 ± 12				
**Average**	265 ± 22	27.6 ± 6.9	−104 ± 19				

The average values of the unfolding characteristics are Δ*V*_*u*_ = (−104 ± 6)*ml mol* ^−1^ and $\Delta G_{u,atm}^{0}=\left(27.6\pm 1.7\right)kJ\;mol^{-1}$ resulting in *K*_*u*,*atm*_ = (1.5 ± 1.0) × 10^−5^. Thus, the unfolding process is highly unfavourable at atmospheric pressure but can be induced by the high pressure of more than 200 MPa. It agrees with the NMR study by Ishima et. al. [27] who observed the existence of folded monomers of HIV-1 PR, as well as with observations of folded monomers of different retroviral proteases [28,33].

**Fig 9:**
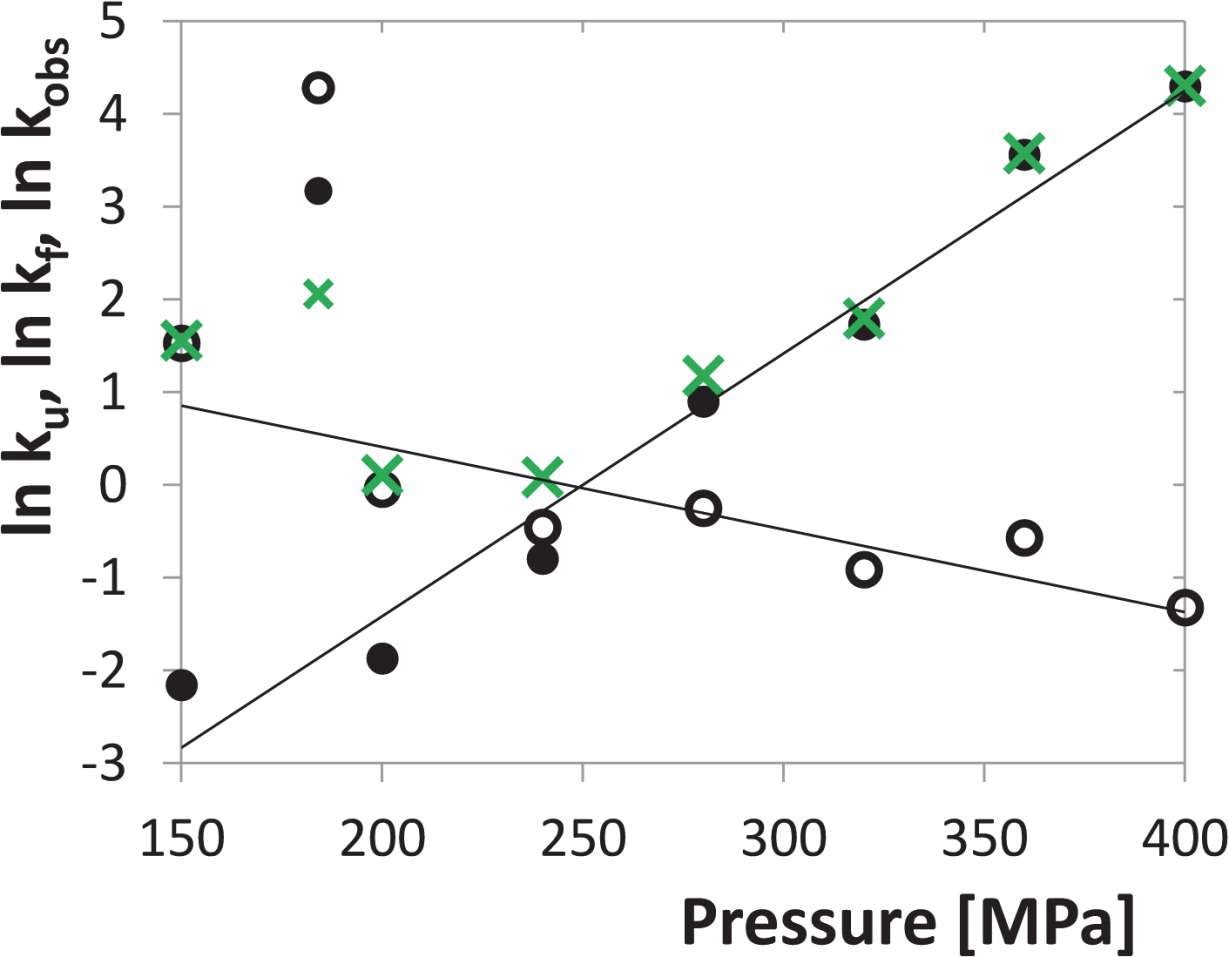
Individual rates of folding and unfolding of the protease monomers and the observed relaxation rate for the concentration of 5 μM dimer. Both rates are fitted by linear functions. The crossing point roughly corresponds with the inflex point of the unfolding transition. Rate constants are considered as dimensionless quantities related to the units of h^−1^.

The values of the relaxation rate constant *k*_*obs*_ (*p*) were determined for all the pressures and the rate constants *k*_*f*_ (*p*) and *k*_*u*_ (*p*) were calculated using Eqs. (13–15). Fig. 9 shows linear dependences of the folding and unfolding rate constants, while the relaxation constant *k*_*obs*_ (*p*) has a minimum in the crossing point of the former lines where the equilibrium constant equals 1. This feature indicates microreversibility of the transition. Finally, the apparent activation volume change and the atmospheric pressure rate constants *k*_*f*,*atm*_ and *k*_*u*,*atm*_ were determined using linear regression of the data by the functions

$$\text{ln}\;k_{i}\left(p\right)=\text{ln}\;k_{i,atm}-\frac{\Delta p\Delta V_{i}^{\neq }}{RT},$$ (21)

where “*I*” stands for “*u*” or “*f*” and $V_{i}^{\neq }$ is the activation volume of the respective reaction. The values for 2 and 5 μM dimer are shown in Table 1. It is obvious that these values, especially the rate constants, are strongly pressure dependent which might be a consequence of the dimerization influence on the unfolding process. Thus, these parameters cannot be considered as reliable characteristic of the unfolding itself, but as an evidence of the mutual interplay of unfolding and dimerization.

Unfolding of monomers influences also the reversibility of the CSM transition assigned to the dimer dissociation. Reversed transition curves measured along with the release of the pressure from the highest value down to atmospheric pressure are shown in Fig. 6 together with the original transition curves. They approximately follow the trend of the original curves, but they are variously shifted for different concentrations. The protease monomers unfold at about 264 MPa in a reversible, but slow process, which can start only after dissociation of the dimer. Therefore, for the lower concentrations of 1–5 μM the recovery of CSM is delayed by folding of the unfolded monomers that are not able of dimerization. The weakening of this effect from 1 to 5 μM might be explained by the longer time the protein spent in the monomeric form for the lower concentrations at which the dimer dissociates at lower pressure. At the higher concentrations of 12 and 25 μM the recovery curve has the expected shape but is shifted up, most likely due to the contribution of protein aggregation occurring at this concentration range (see section “Protein aggregation” below).

### High-pressure behaviour of HIV-1 protease indicated by ANS fluorescence

**Fig 10:**
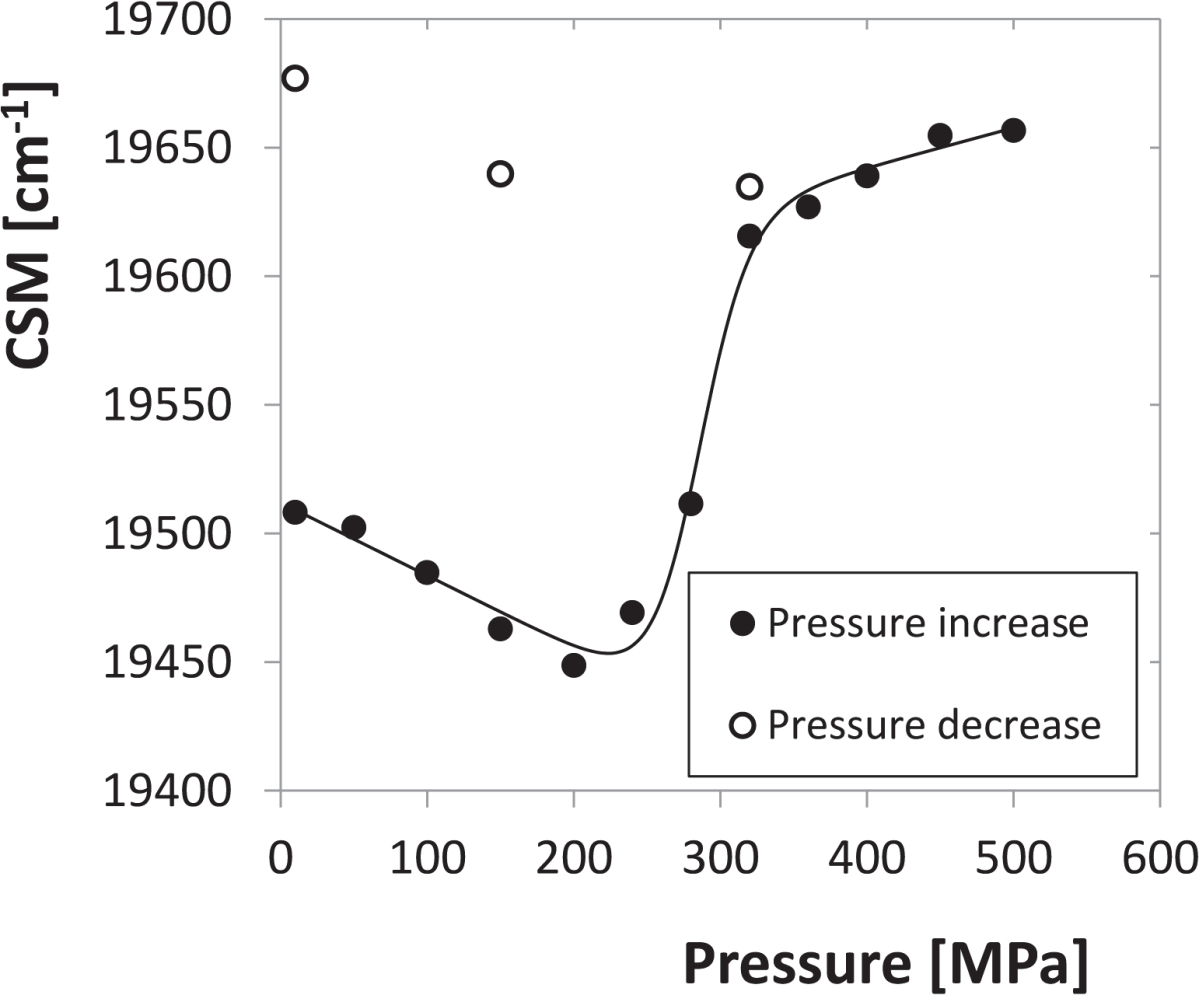
Equilibrium values of CSM of ANS emission spectrum for 10 μM dimer fitted by a model function. Values for the reverse course of pressure indicate irreversibility of the transition.

8-anilino-1-naphthalenesulfonic acid (ANS) is a fluorescent indicator that binds to proteins, mainly by hydrophobic interactions. This interaction is accompanied with a fluorescence increase and a blue shift of ANS emission spectrum, which can be used to indicate the structural changes of proteins, especially the unfolding [34]. A series of fluorescence spectra under various pressures was measured as described in Methods and Material. Up to 240 MPa only a continuous decrease of the CSM with growing pressure occurs without any sign of a transition. Above this pressure, a remarkable change in the spectral shape takes place due to the increased emission at 472 nm which shifts CSM steeply up. The time course of this transition was investigated by pressure-jump experiments. A time series of emission spectra was taken at each pressure point and the pressure dependence of CSM was fitted by Eq. (19). The apparent equilibrium values were plotted vs. pressure (Fig. 10) and the transition parameters were determined as in the previous section. The values of apparent Δ*G*_*ANS*,*atm*_ = (43.5 ± 5.8)*kJ mol* ^−1^ and Δ*V*_*ANS*_ = (−160 ± 21)*ml mol* ^−1^ confirm a transition strongly unfavourable at pressures up to 240 MPa with atmospheric-pressure equilibrium constant *K*_*ANS*,*atm*_ = 2.4 × 10^−8^, but highly favourable above 320 MPa. Above this pressure the shift of equilibrium CSM value is small indicating that the transition runs almost to completeness. Stepwise release of the pressure did not lead to the return of the fluorescence intensity to the value before the transition. Hence, this transition is either irreversible or the kinetics of the reverse process is very slow.

The observed relaxation rate of this transition is almost pressure independent above the inflex point at about 270 MPa. At 320 MPa, where the apparent equilibrium is strongly shifted to the unfolded state, the observed relaxation rate constant *k*_*obs*,*ANS*_ = (0.040 ± 0.003)*min* ^−1^ which gives an unfolding half time *τ*_1/2,*ANS*_ = (17.5 ± 1.23)*min*.

The spectral properties of this transition show no significant concentration dependence which indicates a first-order transition. For low concentration of 2 μM dimer almost no transition-like shape was observed (data not shown), most likely due to the low spectral response of ANS at low protein concentration.

### Protein aggregation

At protein (dimer) concentrations above 10 μM a dramatic increase of both the CSM and the spectral intensity was observed at pressures above 300 MPa. It is demonstrated on both the raw and differential spectra in S1 Fig. and S2 Fig. The steepness of the respective part of the transition curves grows dramatically with protein concentration, while the point where this effect starts tends to lower pressure. Figs. 1 and 3 show this effect especially for 12,5 and 25 μM dimer and the difference in steepness and position shift is obvious. It indicates that, in contrast to the monomer-dimer equilibrium, in this process the total volume decreases along with the decreasing number of particles. Most probably this transition represents pressure-induced protein aggregation. Formation of higher aggregates agrees with the CSM increase (i.e. shift to lower wavelengths) as the spectrum is partially contaminated by the elastically scattered excitation light. Indeed, at the highest concentration the aggregates were visible after the experiment in the sample cuvette as white opacity.

### Enzyme kinetics of HIV-1 protease

**Fig 11:**
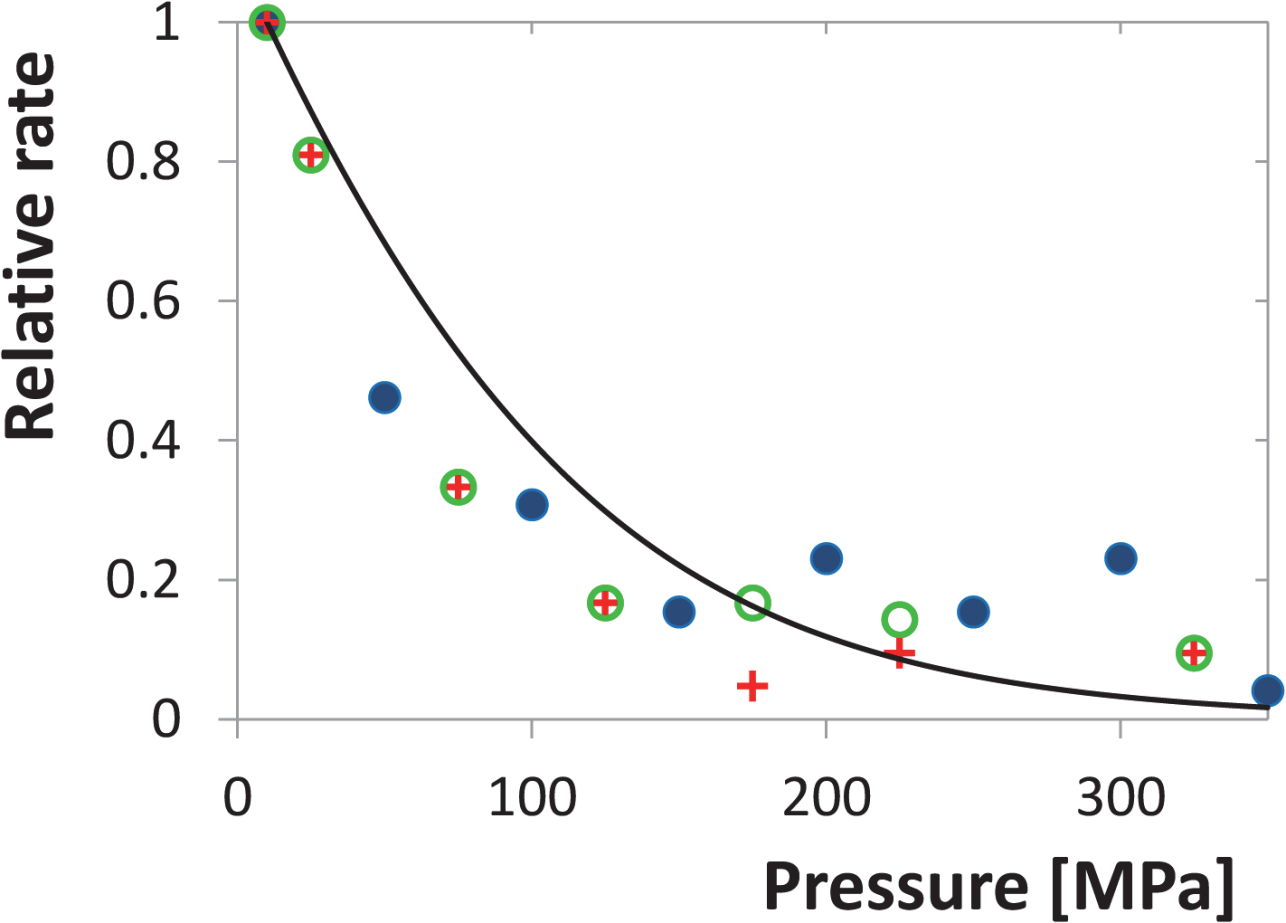
Initial rate of substrate cleavage as a function of pressure related to the value for 10 MPa. Different symbols denote three experimental series. The solid curve shows the relative rate for the ideal case of pressure dependent *K*_*d*_ but pressure independent *K*_*m*_ and *k*_*cat*_.

Enzyme kinetics was investigated under the varying pressure in the region of 10–350 MPa. The substrate was mixed in a spectrophotometric cuvette with the reaction buffer and enzyme and this sample was transferred immediately to the high-pressure cell of the instrument. As this manipulation requires time in the order of seconds to minutes, the concentrations were chosen in order that the linear decrease of substrate concentration takes considerably longer time. The measured reaction rate was then normalised to the initial value at 10 MPa and plotted vs. pressure (see Fig. 11). The resulting curve is decreasing steeply but the activity remains detectable up to 350 MPa. The identification of the individual phenomena contributing to this decrease is rather difficult. The general function describing the rate of the enzyme reaction, which can be derived from the ordinary Michaelis-Menten kinetics and the monomer-dimer equilibrium (Eq. 3), is

$$v=k_{cat}\frac{S}{K_{m}+S}\left[D_{0}+\frac{K_{m}K_{d}}{8\left(K_{m}+S\right)}\left(1-\sqrt{1+\frac{16D_{0}\left(K_{m}+S\right)}{K_{m}K_{d}}}\right)\right].$$ (22)

However, in principle all the three parameters, *K*_*m*_, *K*_*d*_ and *k*_*cat*_ are pressure dependent, but the dependence is known to us only for *K*_*d*_. Determination of this function for the other two quantities would require measurements at different enzyme concentration, which was impossible on the used equipment. In addition, the volume changes that determine the pressure dependencies of the parameters need not be constant. Finally, considering reasonable values of volume changes, all the parameters may influence the reaction rate in a quantitatively comparable way. Nevertheless, considering the pressure-independent behaviour of the inhibited enzyme, it is conceivable that the interaction of enzyme and substrate will behave similarly and that *K*_*m*_ and *k*_*cat*_ will be less pressure dependent. The solid curve in Fig. 11 shows the model situation if only *K*_*d*_ is pressure dependent with the previously determined values of Δ*V*_*d*_ and *K*_*d*,*atm*_. The curve obviously follows the trend of experimental points, but there are some deviations, especially at the high-pressure region, which may be attributed to the pressure dependence of *K*_*m*_ and *k*_*cat*_.

## Discussion

HIV-1 protease structure changes have been investigated under high-pressure ranging from 10 to 600 MPa. The transitions between structural states of the enzyme have been monitored with the aid of tryptophan and ANS fluorescence spectra.

A pressure-dependent transition that moves to higher pressures with growing concentration was identified with the dissociation of the protease dimer. It is followed by another, concentration independent, transition which can be associated with the unfolding of monomers. The former process seems to be very fast keeping the system in permanent monomer-dimer equilibrium, while the latter one is rather slow with the half-time in the order of tens of minutes.

The former process is reflected by changes in the shape of the tryptophan emission spectrum which can be observed as a difference in the CSM profile for inhibited and non-inhibited enzyme (Fig. 1A). Obviously, the total spectral change consists of a structure-independent contribution of the direct influence of high pressure to the fluorophores that shifts the CSM monotonously to lower values [31] and a contribution depending on dimer dissociation which is missing at the inhibited enzyme. This is apparent also in the differential spectra shown in S2 Fig., especially on the branch where the minima are located. For the higher concentrations of 12 and 25 μM, where the dimer dissociation occurs only at relatively high pressures, the minima of the curves are initially stable at about 320–330 nm, similarly to the inhibited enzyme. (The small difference in their position the inhibited and non-inhibited enzyme is likely caused by the presence of darunavir, which is itself an aromatic molecule.) With growing pressure the position of the minima moves up. On the contrary, for the low concentration of 2 μM, where the dimer dissociation progresses already at the lower pressures, the minimum is initially located approximately at 350 nm and moves down when the pressure grows. It is thus probable that the structure-independent contribution causes an intensity decrease at 320–330 nm while the dimer dissociation at higher wavelength. At high concentration the structure-independent contribution prevails at lower pressures and is supplemented by the dimer-dissociation contribution only in the high-pressure region, while at lower concentrations the dimer-dissociation contribution dominates at low pressures and is overwhelmed by the structure-independent contribution when the dimer dissociation draws to completeness. Thus, for high concentrations the minimum position moves up with increasing trend while for low concentrations they move down with decreasing trend, in all cases tending to approximately equal point between 330 and 340 nm. For the higher concentrations of 12 and 25 μM the high-pressure spectra are probably influenced by protein aggregation which causes the intensity increase at the low-wavelength range as a consequence of the elastic scattering of the excitation light. Obviously, it also changes the position of the differential-spectrum minimum dramatically.

The unfolding process is reflected by the intensity of both tryptophan and ANS fluorescence spectra. The ANS spectral intensity shows a transition at equal pressure as the tryptophan fluorescence, but considerably steeper and with a rather sudden onset above 240 MPa. This may be explained by co-operative action of the monomer unfolding and stabilization of the unfolded state by binding of ANS. Given the values of *K*_*u*,*atm*_ and Δ*V*_*u*_, the degree of unfolding starts to grow rapidly from the point where *K*_*u*_(*p*) = 1. As a consequence, the rate of unfolding increases dramatically in this region. Thus, the unfolding process becomes strongly favoured above the inflex point both thermodynamically and kinetically. As previously discussed, ANS fluorescence provides a sufficient signal only at protein concentration higher than 2 μM. Unfolded protein reaches this concentration closely after the pressure crosses the inflex point. The interaction with ANS can probably further stabilize the unfolded state or change the direction of the process to another unfolding pathway, which may contribute to the irreversibility of this process. A similar process of destabilization of the native structure of human serum albumin by interaction with ciprofloxacin was reported recently [35]. The common base of both the tryptophan- and ANS-indicated transitions can be further supported also by the rough agreement of the relaxation rate constants.

When an inhibitor is added to the system, none of the transitions occurs within the whole pressure range. It can be deduced that the inhibitor stabilizes the dimer structure, as is generally assumed even at atmospheric pressure. These results indicate that the dimer structure is remarkably firm and does not undergo unfolding even at very high pressure. Unfolding transitions have been observed only at the non-inhibited enzyme above about 265 MPa where a substantial part of the dimer was already dissociated, which indicates that unfolding can be initiated only in the monomeric state of the protease.

The high-pressure destabilization of HIV-1 PR dimer allowed us to determine the equilibrium constant of dimer dissociation *K*_*d*,*atm*_, which is a challenging experimental problem for decades. At atmospheric pressure determination of this constant is complicated by the high stability of dimer and requires rather low enzyme concentrations. In contrast with this, high pressure, together with negative Δ*V*_*d*_, destabilizes the dimer and shifts the transition to higher concentrations. This fact can be helpful also for other experimental assays where free monomers are needed, e.g. to confirm their structure by NMR. Furthermore, compounds interacting directly with monomer, especially potential inhibitors of dimerization [36] or modular inhibitors inhibiting both the catalytic activity and dimer formation [37] studied by some research groups in the past, can be designed with the aid of these techniques.

The method of determination of *K*_*d*,*atm*_ presumes that Δ*V*_*d*_ is constant within the whole pressure range. This assumption is supported by the linearity of the dependence of the CSM inflex point vs. pressure depicted in Fig. 5. Eventual deviations from linearity might occur at the lowest part of the pressure range where no experimentally detected inflex points are located, but their influence on the equilibrium constant would be minor, because the reaction Gibbs energy $\Delta G_{d,atm}^{0}$ is an integral of Δ*V*_*d*_ along the pressure axis. Thus, the resulting *K*_*d*,*atm*_ value can be considered to be correct at least as regards the order of magnitude.

The previously reported values of equilibrium constant *K*_*d*,*atm*_ differ by several orders of magnitude among each other. In general, the values obtained by kinetic assays [4–7], including our study [6], are lower than those obtained by substrate/inhibitor-independent assays [9,10,38], which might be caused by an influence of the substrate/inhibitor stabilization. Our current value is higher that the kinetically determined values, but belongs to the lower end of the set of values determined by the substrate/inhibitor-independent methods.

The present results show that the dimer dissociation and association are both fast processes that keep the system in permanent equilibrium. Together with the observed substrate or inhibitor stabilization it means that the dimerization equilibrium is no hindrance of the enzyme activity since the active dimers re-associate even at low concentrations immediately after the addition of the substrate. Previously, rather mixed results have been reported regarding the question of fast or slow dimerization equilibrium. The method of sedimentation equilibrium [9,10,38] could not resolve this question, while the kinetic methods usually reported a slow dimerization process [4,5,7,37,39–41]. However, at atmospheric pressure dimerization can be easily confused with some unfolding process, because the missing pressure coordinate prevents the distinguishing between the first and second order transitions. Although at the current experiment no unfolding processes running at atmospheric pressures have been found, at different conditions, e.g. temperature of 37°C, they may be enabled.

Enzyme-kinetics experiments showed that the enzyme keeps a part of its activity up to 350 MPa. It indicates that also the substrate provides a similar structure stabilizing effect as an inhibitor, although it is continuously cleaved to products. There is, however, a strong decrease of the reaction rate in the pressure interval of 10 to 150 MPa which might be caused by pressure dependences of at least three different parameters, *K*_*m*_, *K*_*d*_ and *k*_*cat*_. It is plausible that the enzyme with bound substrate behaves similarly as with inhibitor and is thus rather inert with respect to pressure. In such a case *K*_*d*_ can be considered as a major source of pressure dependence. Indeed, the experimental results follow the theoretical model with reasonable accuracy, but deviations from it can be observed at the high pressure region where the measured rate exceeds the theoretical assumption. However, to explain this effect, that can be caused by several partial phenomena, further experiments are required that allow us to vary the substrate concentrations in a broad range. Thus, high pressure enzyme kinetics remains an interesting experimental challenge for the future.

## Conclusion

Inhibitor and substrate binding protect HIV-1 PR against dissociation and unfolding. Unfolding of the protein is initiated only from the monomeric state. In the absence of substrate, the protein is not extremely stable, however, the *K*_*d*,*atm*_ in the submicromolar range protects the protein from dissociation and unfolding at atmospheric pressure and 25°C. Unfolding processes occur only at pressures above 250 MPa. High pressure destabilizes the dimer structure, which opens an interesting way for experiments requiring higher population of monomers in the system. It can facilitate studies of the process of dimerization, structure of monomers and their interaction with different compounds which can be used for design of potential dimerization inhibitors.

## Supporting Information

S1 Fig Selected tryptophan emission spectra of HIV-1 PR in the concentration of 25 μM dimer. (PDF)

S2 Fig Sets of differential tryptophan-emission spectra for different concentrations of HIV-1 PR dimer. For 12 μM spectra for inhibited and non-inhibited enzyme are shown. Each spectrum is fitted by 4^th^-order polynomial in order to identify the changes in the shape of the spectrum and positions of its extremes. For the method of calculation of these spectra see Methods and materials, section “High pressure fluorescence experiments”.
(PDF)

S3 Fig Reversibility assay of HIV-1 PR of 5 μM concentration (expressed as dimer). For every enzyme sample three CSM values were determined: at 10 MPa before pressurizing, at the selected target pressure from the interval of 100 to 350 MPa 3 min after setting the pressure (in contrast to an analogous experiment shown in Fig. 6 where the incubation time was 50 min) and again at 10 MPa after releasing the pressure.
(EPS)
