# Supplementary figures and images for: Inhibitor and Substrate Binding Induced Stability of HIV-1 Protease against Sequential Dissociation and Unfolding Revealed by High Pressure Spectroscopy and Kinetics
Source: PLoS One. 2015 Mar 17;10(3):e0119099. doi: 10.1371/journal.pone.0119099 (PMC4362767; doi:10.1371/journal.pone.0119099)

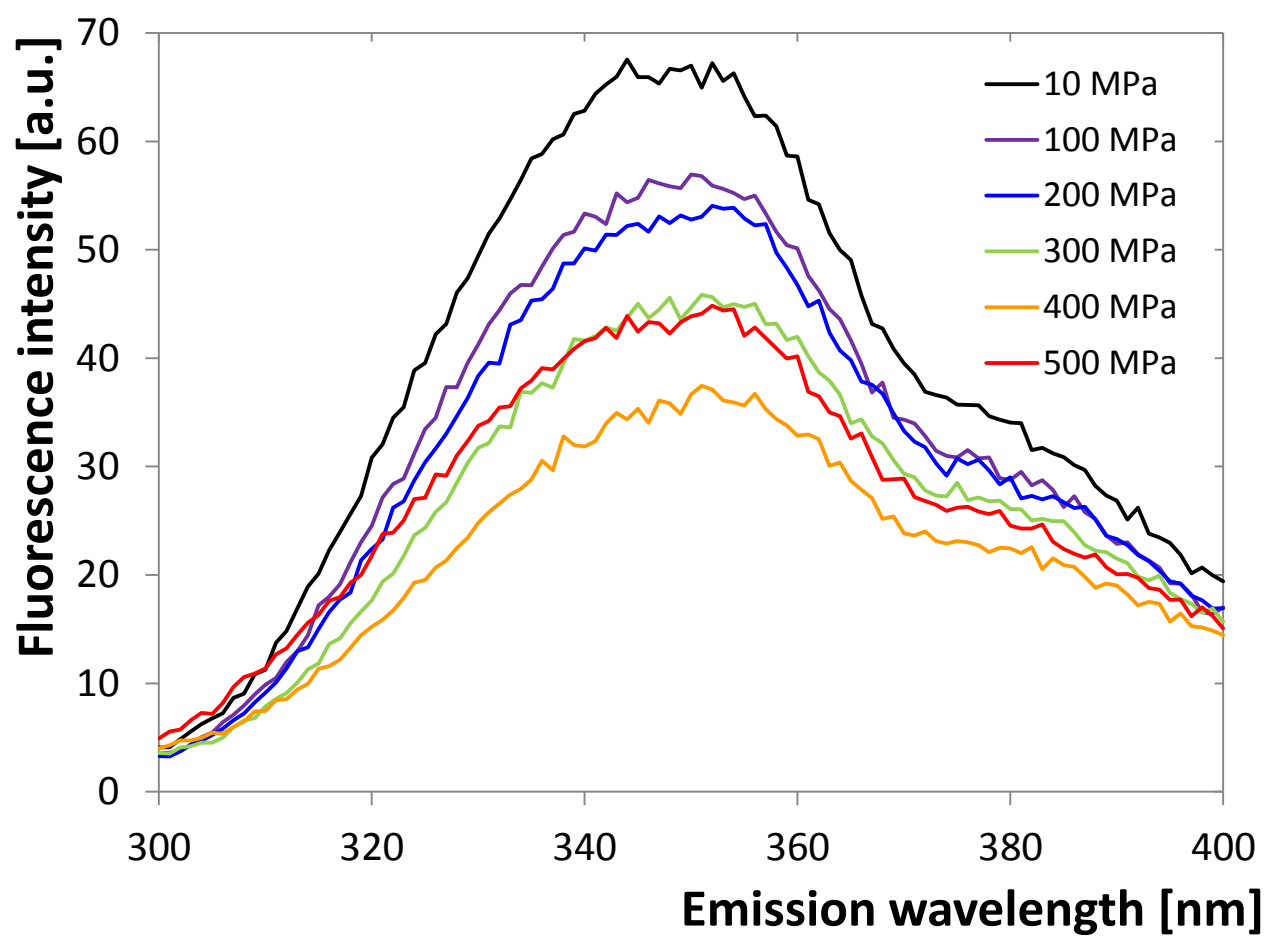

Supplement: S1 Fig — (PDF) [file pone.0119099.s001.pdf]

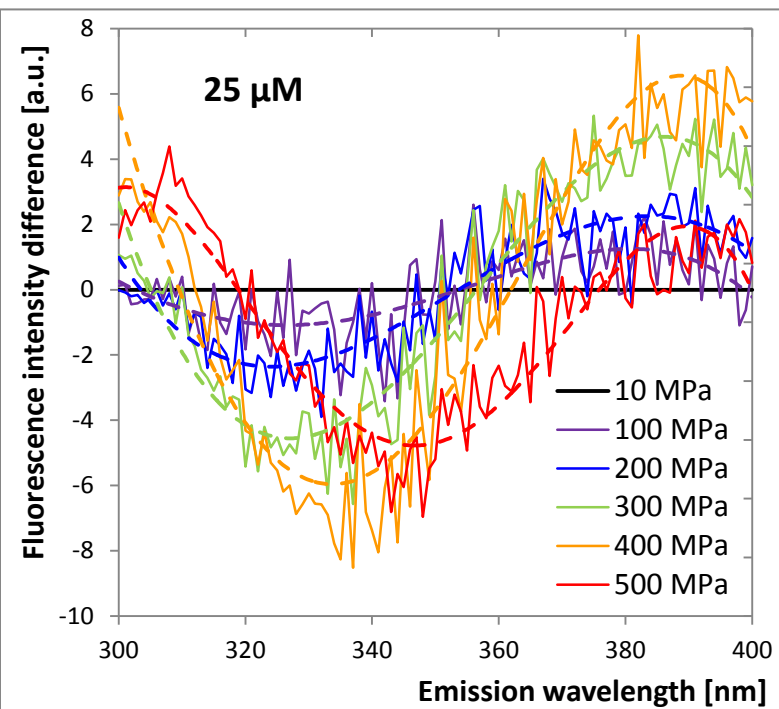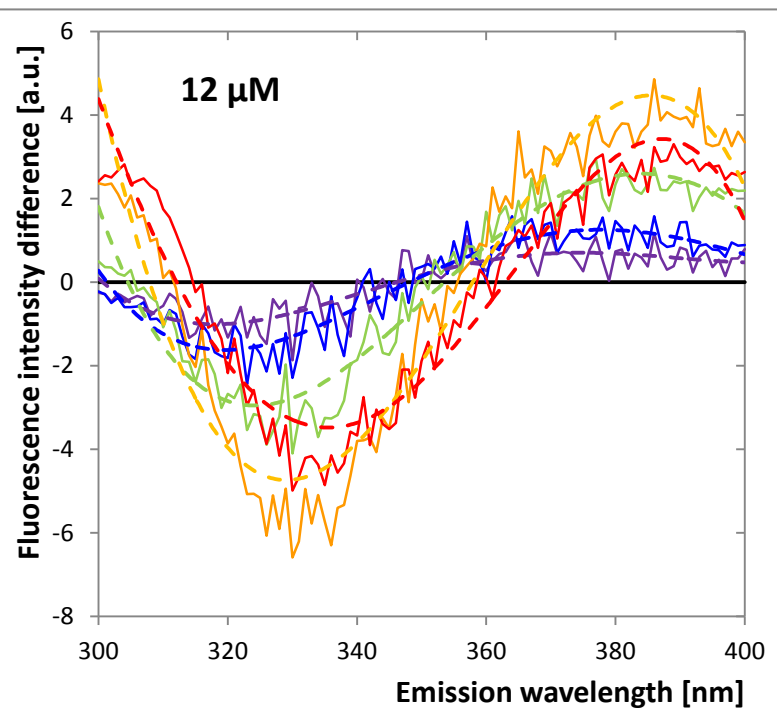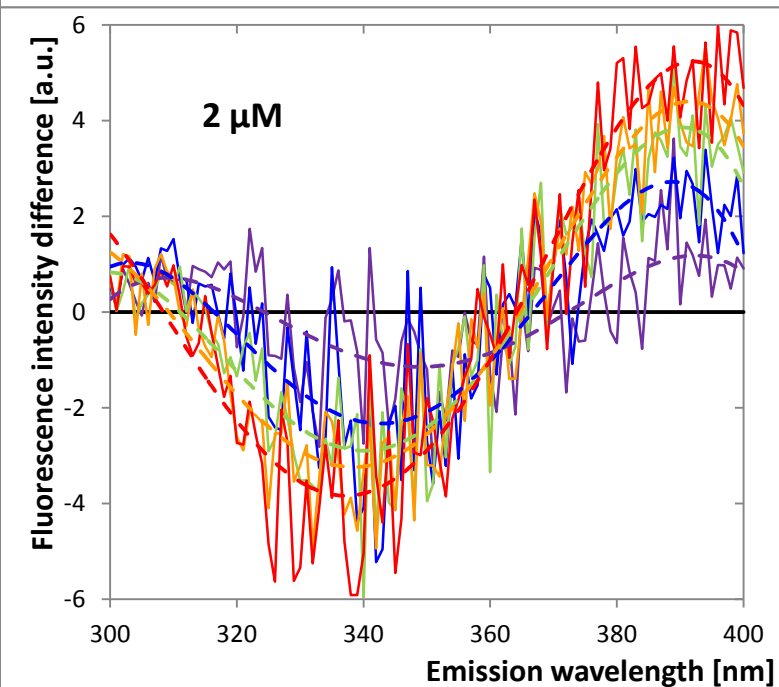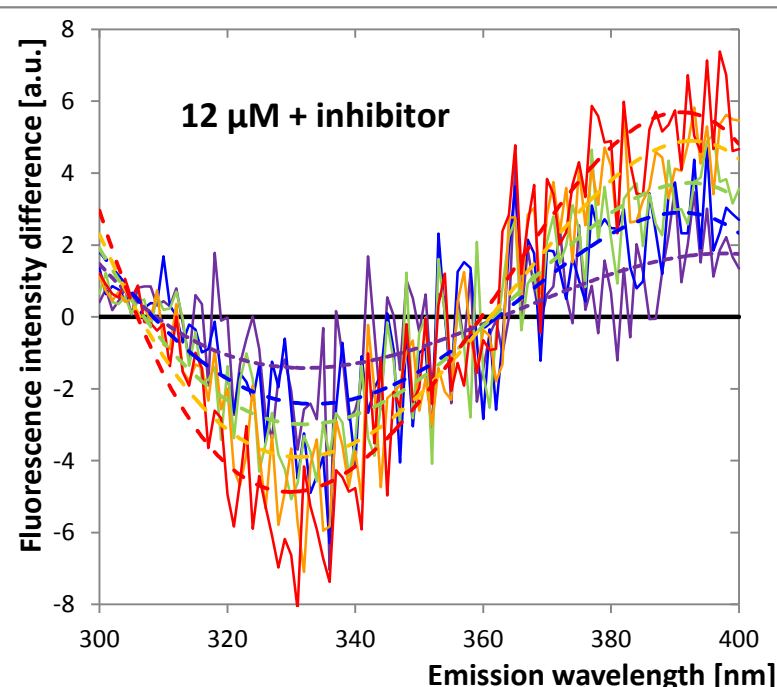

Supplement: S2 Fig — For 12 μM spectra for inhibited and non-inhibited enzyme are shown. Each spectrum is fitted by 4th-order polynomial in order to identify the changes in the shape of the spectrum and positions of its extremes. For the method of calculation of these spectra see Methods and materials, section “High pressure fluorescence experiments”. (PDF) [file pone.0119099.s002.pdf]
